# *p53 *tumor suppressor gene mutations in fibroblast-like synoviocytes from erosion synovium and non-erosion synovium in rheumatoid arthritis

**DOI:** 10.1186/ar1448

**Published:** 2004-10-29

**Authors:** Yuji Yamanishi, David L Boyle, Douglas R Green, Edward C Keystone, Alison Connor, Susan Zollman, Gary S Firestein

**Affiliations:** 1 Department of Rheumatology, Hiroshima City Hospital, Hiroshima, Japan; 2 Division of Rheumatology, Allergy, and Immunology, School of Medicine, University of California at San Diego, La Jolla, California, USA; 3 La Jolla Institute of Allergy and Immunology, La Jolla, California, USA; 4 Department of Medicine, University of Toronto, Canada

**Keywords:** erosion, fibroblast-like synoviocytes, invasiveness, p53 mutation, rheumatoid arthritis

## Abstract

Abnormalities in the *p53 *tumor suppressor gene have been detected in rheumatoid arthritis (RA) and could contribute to the pathogenesis of chronic disease. To determine whether synoviocytes from invasive synovium in RA have an increased number of mutations compared with non-erosion synoviocytes, *p53 *cDNA subclones from fibroblast-like synoviocytes (FLS) derived from erosion and non-erosion sites of the same synovium were examined in patients requiring total joint replacement. Ten erosion FLS lines and nine non-erosion FLS lines were established from nine patients with RA. Exons 5–10 from 209 *p53 *subclones were sequenced (114 from erosion FLS, 95 from non-erosion FLS). Sixty percent of RA FLS cell lines and 8.6% of the *p53 *subclones isolated from FLS contained *p53 *mutations. No significant differences were observed between the erosion and non-erosion FLS with regard to the frequency or type of *p53 *mutation. The majority of the mutations were missense transition mutations, which are characteristic of oxidative damage. In addition, paired intact RA synovium and cultured FLS from the same joints were evaluated for *p53 *mutations. Matched synovium and cultured synoviocytes contained *p53 *mutations, although there was no overlap in the specific mutations identified in the paired samples. Clusters of *p53 *mutations in subclones were detected in some FLS, including one in codon 249, which is a well-recognized 'hot spot' associated with cancer. Our data are consistent with the hypothesis that *p53 *mutations are randomly induced by genotoxic exposure in small numbers of RA synoviocytes localized to erosion and non-erosion regions of RA synovium. The determining factor for invasiveness might be proximity to bone or cartilage rather than the presence of a *p53 *mutation.

## Introduction

Rheumatoid arthritis (RA) is a chronic inflammatory disease characterized by synovial tissue proliferation with progressive joint destruction. The etiology of RA remains unknown, but many factors, including autoimmunity, cytokines and genetic factors, participate in its pathogenesis [1,2]. Although inflammation and joint destruction can be intimately related, the two processes might also be independent in some circumstances [3,4]. This observation might be explained, at least in part, by autonomous behavior by fibroblast-like synoviocytes (FLS) [5]. These cells exhibit some features of transformation in RA, including loss of contact inhibition, anchorage-independent growth, oncogene activation, autonomous invasion into cartilage and somatic gene mutations [4,6-9]. One potential gene that might contribute to this phenotype is the *p53 *tumor suppressor gene, which plays a critical role in cell-cycle regulation, DNA repair, senescence, genomic stability and apoptosis [10]. *p53 *is expressed in many inflammatory and autoimmune diseases [11-13], and it may serve a protective function by suppressing cytokine production and matrix destruction [14,15]. For instance, mice lacking the *p53 *gene have significantly greater joint destruction compared with wild-type controls in the collagen-induced arthritis model [16].

The function of *p53 *can be altered through somatic mutations in both neoplasia and non-malignant conditions such as ulcerative colitis, sun-exposed skin and chronic RA [8,17,18]. The mutations in RA synovium reside primarily in the intimal lining and have also been identified in cultured FLS, which are thought to originate from this region [8,9]. *p53 *sequencing studies in RA have until now focused on synoviocytes derived from non-erosion regions of the synovium that are readily obtained at the time of joint replacement surgery. Because some reports suggest that monoclonal expansion and oligoclonal expansion of synoviocytes occur at sites of invasion [19], we evaluated the relative frequency of *p53 *mutations in FLS in paired erosion and non-erosion synoviocytes from the same patients. Our data suggest that mutations are present in both regions and that the tendency to invade may be related to proximity to the extracellar matrix.

## Materials and methods

### Synovial tissues and preparation of FLS

Synovial tissue samples were collected with informed consent at the time of joint replacement from patients with RA. The diagnosis of RA conformed to the 1987 revised American College of Rheumatology criteria [20]. Separate samples were obtained, at the University of Toronto, from within erosions at the periphery of the articular surface and from non-erosion sites collected from the intracapsular non-articular surface. Erosion FLS lines and non-erosion FLS lines were then prepared from each patient.

FLS cell lines were established as previously described [21]. Briefly, tissues were minced by sterilized scissors, and were incubated with 1 mg/ml collagenase in serum-free DMEM for 2 hours at 37°C, were filtered through a nylon mesh, were extensively washed, and were cultured in DMEM containing 10% fetal calf serum, 2 mmol/l glutamine, 50 μg/ml gentamicin, 100 U/ml penicillin, and 100 μg/ml streptomycin, in a humidified 5% CO_2 _atmosphere. After overnight culture, cells were trypsinized, split in a 1:3 ratio, and were recultured in medium. FLS from passages 5–8 were used in these experiments, during which time they represented a homogeneous population of FLS (< 1% CD 11b, < 1% phagocytic, and < 1% Fc-gamma RII receptor-positive). A second set of FLS samples obtained from the University of California at San Diego were obtained with a matched sample of synovium, which was embedded in TissueTek OCT compound (Miles Diagnostics, Elkhart, IN, USA), snap frozen, and stored at -80°C until use. Frozen tissues with approximate area of 10 mm^2 ^were cut into 10 μl sections at the time of PCR analysis for *p53 *mutations.

### Production of immunoreactive MMP-1

FLS were cultured to near confluence in six-well tissue culture plates (Falcon, Bedford, MA, USA). IL-1β (3 ng/ml) or medium was added to the wells and was incubated for 72 hours at 37°C in a humidified 5% CO_2 _atmosphere. Supernatants were collected and MMP-1 concentrations were determined by ELISA according to the manufacturer's instructions (Total MMP1Biotrak; Amersham Biosciences, Piscataway, NJ, USA) [22].

### Statistical analysis

Comparisons between two groups were analyzed by the Wilcoxon signed rank test. *P *< 0.05 was considered significant.

## Results

### *p53 *mutations in erosion FLS and non-erosion FLS

To determine whether invasive synovium in RA has an increased number of mutations, *p53 *cDNA subclones from FLS derived from erosion sites and non-erosion sites were examined. Ten erosion FLS lines and nine non-erosion FLS lines were established from nine patients with RA (two erosion lines and one non-erosion line were examined in one patient). A total of 209 *p53 *subclones were subjected to sequence analysis (114 from erosion FLS, 95 from non-erosion FLS), and *p53 *exons 5–10 were examined. As shown in Table 1, *p53 *mutations were identified in 11 out of 19 FLS lines (four of 10 erosion FLS lines, and seven of nine non-erosion FLS lines). Eighteen subclones out of the total 209 (8.6%) contained mutations. There were no significant differences in the frequency of *p53 *mutations between erosion FLS and non-erosion FLS (7.9% and 9.5%, respectively). Nested PCR was required for one of the FLS lines (RA4 non-erosion FLS line). The rate of mutation with this line was similar to the other erosion and non-erosion FLS.

**Figure 1:**
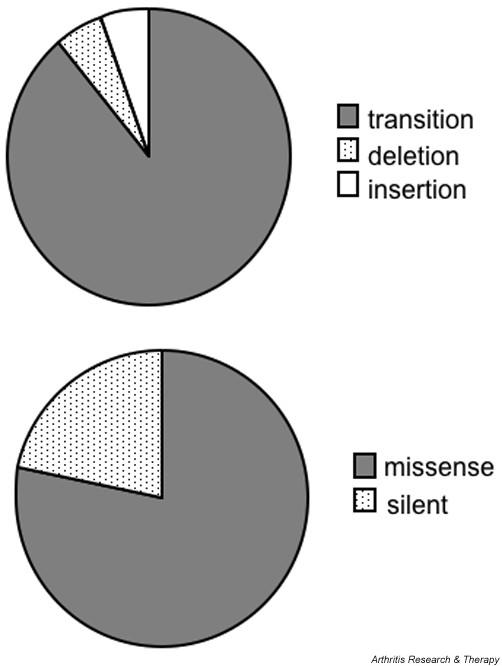
Types of *p53 *mutations in rheumatoid arthritis fibroblast-like synoviocytes (FLS). Because no differences were observed between erosion FLS and non-erosion FLS (see Table 1), results were pooled.

As in previous reports [8,9,23], most *p53 *mutations (eight of nine in erosion FLS and eight of nine in non-erosion FLS) were transition mutations (i.e. G>A or C>T), which are characteristic of mutations caused by oxidative damage [24,25], and no transversion mutations (i.e. G>T, A>T, C>A, C>G) were seen (see Fig. 1 for the pooled data). One single base deletion and one multinucleotide insertion were detected. The majority of *p53 *mutations (78%) were missense (see Fig. 1 for pooled data). Most of the mutations were identified in a single subclone, although multiple copies of one mutation in codon 321 AAA to GAA were observed in an erosion FLS line. These data suggest that the frequency and types of mutations are similar in FLS isolated from either sites of erosion or from regions that are not invading into bone or cartilage.

### MMP-1 expression in erosion and non-erosion FLS

Two matched erosion and non-erosion FLS lines were also evaluated for medium-stimulated and IL-1β-stimulated collagenase gene expression (MMP-1). There were no differences between the erosion or non-erosion samples with regard to either basal or cytokine-stimulated MMP-1 protein concentrations in the culture supernatants (basal, 5.5 ± 1.2 ng/ml; IL-1 stimulated, 24.4 ± 3.2 ng/ml).

### *p53 *mutations in matched RA FLS and synovial tissue samples

After evaluating the matched FLS lines, we then examined the mutations in whole RA synovium and FLS isolated from the same joint. The matched erosion and non-erosion synovia from the preceding analysis were not available, so subclones from four additional paired RA FLS and synovial tissues from non-erosion regions were sequenced for *p53 *mutations (see Table 2). Mutations were detected in 12 of the 43 *p53 *subclones from RA FLS (28%), and in five of 46 subclones from the paired RA synovial tissues (11%). The relatively higher percentage of mutations in this limited number of lines compared with those presented in Table 1 is within the range observed for RA FLS in other studies. A few of the subclones contained two mutations (two of the RA FLS subclones, and one of the RA tissue subclones). Distinct patterns were found in the RA FLS compared with the paired tissue *p53 *subclones (see Table 2), although the frequency of mutations was somewhat higher in these samples compared with those presented in Table 1 and previous reports [8,9,23].

Mutation analysis of FLS revealed eight transitions, three transversions, and three deletions among 14 mutations in RA FLS. Of the base changes, 11 were missense and three were silent (Table 2). The RA tissue samples had five transition mutations (four A>G, one C>T) and one transversion mutation (G>T), with four mutations identified as missense and two as silent. Nested PCR was required for two FLS lines, RA11 and RA12 FLS, and these results were similar to the cell lines that did not require nested PCR. RA13 synovial tissue had no mutations identified despite the use of nested PCR, indicating the fidelity of this process.

Interestingly, FLS from one patient had multiple subclones containing a mutation at codon 249 (AGG>GGG [Arg>Gly]) (see Table 2). Codon 249 missense mutations have been detected frequently in malignant tissues [26,27]. In another patient, a silent codon 213 base change (CGA>CGG [Arg>Arg]) was detected in 50% of *p53 *subclones from both FLS and synovial tissues. This same base change was also present in peripheral blood mononuclear cells of the patient (data not shown) and probably represents a known *p53 *germline polymorphism [28].

## Discussion

The aggressive nature of RA synovium and the ability of cultured synoviocytes to invade autonomously in cartilage suggest that these cells might be permanently altered or imprinted by their sojourn through the rheumatoid joint [4,5]. Additional data evaluating expression of X-linked genes indicate that oligoclonal or monoclonal expansion of synoviocytes can occur in chronically inflamed rheumatoid synovial tissue, especially at sites of erosions [19]. More recently, we showed that islands of cells expressing mutant *p53 *genes are present in the rheumatoid synovial intimal lining and that these regions produce significantly higher amounts of IL-6 [9]. However, the relationship between mutations and the synovial invasion has not been systematically examined.

In the present study, we first examined paired samples of synoviocytes isolated from the erosive front of synovium and from regions not directly adherent to bone or cartilage for *p53 *mutations. Sixty percent of the cell lines examined had mutations, and 8.6% of the subclones isolated from either site contained mutations. No significant differences were observed between erosion and non-erosion FLS with regard to the frequency or type of mutation. Previous reports describe *p53 *base changes in 15–50% of RA FLS lines, with a frequency of mutations within the *p53 *cDNA pool varying from 0% to 30% [8,23,29]. The broad range might relate to the methods used to identify mutations, some of which are less sensitive (e.g. single-strand conformation polymorphism), or may be due to the evaluation of different numbers of exons. Other investigators failed to find mutations, perhaps because the experiments focused on sequencing the unfractionated *p53 *cDNA pool rather than subclones, on evaluation of less severe disease, or on sequencing a limited number of subclones [30,31].

The mutations observed in this study are mainly transition missense base changes, as previously described [8,9,23]. These are characteristic of oxidative deamination by nitric oxide or oxygen radicals [24,25] and are consistent with the hypothesis that the *p53 *mutations are caused by oxidative stress in the inflammatory environment, although this still has not been proven [32]. Relaxation of the DNA mismatch repair mechanisms in synoviocytes also probably contributes to susceptibility to DNA damage. For instance, suppression of *hMSH6 *expression in RA synovium has been associated with synovial microsatellite instability [33]. The majority of mutations in the present study were only identified in individual subclones. However, multiple copies were detected in the sequenced subclones from three patients. One of these at codon 249 is a well-recognized 'hot spot' associated with lung cancer and hepatocellular carcinoma [26,27]. Inazuka and colleagues previously identified another 'hot spot' codon 245 transition mutation in RA FLS lines from two patients [23]. Table 3 summarizes *p53 *mutation clusters (i.e. detected in more than one subclone) in the present study and in the literature in cultured RA FLS or synovial tissue. More than 90% of the repeat *p53 *mutations are missense and have been frequently detected in malignant tissues.

Nishioka and colleagues demonstrated oligoclonal expansion or monoclonal expansion of synoviocytes at the sites of erosion in RA [19]. Furthermore, p53 expression tends to be greatest at sites of invasion in the severe combined immunodeficiency mouse model where cultured FLS erode into cartilage explants [34]. We expected to see an increased number of mutations at these sites as well. However, there were no differences between matched erosion and non-erosion FLS with regard to the number or types of *p53 *mutations. The mutations in very late stage of disease are therefore equally abundant in all regions of the rheumatoid synovium. Although they are clearly present at the invasive front, which might contribute to local tissue destruction, they are not over-represented compared with other sites that have been exposed to the genotoxic environment for the same period of time. The lack of association between invasion into bone and p53 mutations is consistent with recent data suggesting that osteoclasts, rather than synoviocytes, are the primary mediators of bone erosions [35]. FLS might play a more important role in cartilage damage through the elaboration of proteolytic enzymes and cytokines, which are increased in cells lacking functional p53 protein. Mutations in erosion had unique sequences when compared with the paired non-erosion mutations from the same patient, which may not be surprising given the results of microdissection studies demonstrating multiple independent islands of mutant cells rather than diffuse monoclonal expansion [9].

In addition to studying paired erosion and non-erosion FLS, we analyzed a second set of samples where we had the opportunity to examine paired whole non-erosion synovium with FLS isolated from the same joint. Mutations were identified in the matched samples, with similar ratios of transitions and missense changes between cell lines and tissues. There was no overlap in the specific base changes. Because cells in the lining form islands with oligoclonal expansion of individual mutations, cell lines derived from a different fragment of synovium would be expected to have different mutations compared with another region. The percentage of cDNA subclones with mutations was higher in the FLS than in matched synovium. This is most probably because the cells bearing mutations in the intact tissue (synoviocytes in the intimal lining) represent only about 20% of the total compared with 100% of the cells in the homogeneous cell cultures.

Our data are consistent with the proposed hypothesis that *p53 *mutations are randomly induced by genotoxic exposure in small numbers of RA synovial lining cells in both erosion and non-erosion regions. Based on the association of these same mutations with neoplasia and our previous studies showing that these mutations can be dominant negative [36], it is reasonable to suggest that some of the *p53 *mutant cells in RA have selective growth advantage and thus form clusters in RA synovial tissue. Subsequently, the islands can influence cells in the environment through the elaboration of cytokines and factors that are normally suppressed by wild-type *p53 *(such as IL-6). A careful evaluation of erosion and non-erosion sites suggests that cells in both regions are equally likely to contain mutant cells, and that the expression of proteins related to matrix invasion, like MMP-1, was similar in the two cell populations. The determining factor for invasiveness might be proximity to bone or cartilage rather than the presence of a *p53 *mutation. Hence, cells directly adjacent to the matrix can potentially adhere and invade, whereas those cells in non-erosion regions would only have an indirect influence on destruction.

## Conclusions

Mutations of the *p53 *tumor suppressor gene were present in synoviocytes isolated from both erosion and non-erosion sites in longstanding RA. Clusters of mutations can occur in RA synovium, but the abnormal clones are not unique to sites of joint destruction. The determining factor for invasiveness might be proximity to bone or cartilage rather than the presence of a *p53 *mutation. Hence, cells directly adjacent to the matrix can potentially adhere and invade whereas those cells in non-erosion regions would only have an indirect influence on destruction.

## Abbreviations

DMEM = Dulbecco's modified Eagle's medium; ELISA = enzyme-linked immunosorbent assay; FLS = fibroblast-like synoviocytes; IL = interleukin; MMP-1 = matrix metalloproteinase-1; PCR = polymerase chain reaction; RA = rheumatoid arthritis

## Competing interests

The author(s) declare that they have no competing interests.

## Authors' contributions

Yuji Yamanishi designed and executed the study and prepared the manuscript. Edward Keystone, Alison Connor, and Susan Zollman developed erosion and non-erosion FLS. David Boyle assisted with the design of experiments and obtained UCSD clinical samples. Douglas Green evaluated and interpreted data and assisted with preparation of the manuscript. Gary Firestein supervised the project, evaluated and interpreted data, and prepared the manuscript.

## Figures and Tables

**Table 1:** *p53 *mutations in matched erosion fibroblast-like synoviocytes (FLS) and non-erosion FLS from rheumatoid arthritis(RA) patients

Patient	Erosion FLS		Non-erosion FLS	
	
	Mutation	Frequency	Mutation	Frequency
RA1 (two lines examined)				
Line 1	Codon 318 CCA>TCA (Pro>Ser)	1/12	None	0/11
Line 2	None	0/11		
RA2	Codon 194 CTT>TTT (Leu>Phe)	1/13	Codon 274 GTT>ATT (Val>Ile)	1/9
	Codon 226 GGC>GAC (Gly>Asp)	1/13		
RA3	None	0/13	Codon 186 GAT>AAT (Asp>Asn)	1/12
RA4	Codon 155 ACC>GCC (Thr>Ala)	1/12	Codon 266 GGA>AGA (Gly>Arg)	1/12
	Codon 321 AAA>GAA (Lys>Glu)	2/12		
RA5	None	0/10	None	0/11
RA6	None	0/9	Codon 276 GCC>GTC (Ala>Val)	1/7
RA7	None	0/11	Codon 167 CAG>CAA (Gln>Gln)	1/10
			Codon 312 ATC>ATT (Ile>Ile)	1/10
RA8	Codon 212 TTT>TTC (Phe>Phe)	1/12	Codon 176 TGC>TAC (Cys>Tyr)	1/12
	Codon 309 CCC>CCT (Pro>Pro)	1/12	Codon 239 AAC>AGC (Asn>Ser)	1/12
	Codon 317 CAG>AG (deletion)	1/12		
RA9	None	0/11	Codon 261-C262 AGT (Ser) insertion	1/11
Total		9/114		9/95

Data presented as number of mutant subclones/total number of subclones analyzed. Nested PCR used for the RA4 non-erosion FLS.

**Table 2:** *p53 *mutations in paired rheumatoid arthritis (RA) fibroblast-like synoviocytes and synovial tissue samples

Patient	Fibroblast-like synoviocytes		Synovial tissue	
	
	Mutation	Frequency	Mutation	Frequency
RA10	Codon 119 GCC>ACC (Ala>Thr)	1/10	Codon 196 CGA>TGA (Arg>stop)	1/12
	Codon 178 CAC>AC (deletion)	1/10		
	Codon 223 CCT>CAT (Pro>His)	1/10		
	Codon 360 GGG>AGG (Gly>Arg)	1/10		
	Long deletion codon 143 – codon 220	1/10		
	Codon 213 CGA>CGG (Arg>Arg)	5/10^a^	Codon 213 CGA>CGG (Arg>Arg)	6/12
RA11	Codon 307 GCA>GCT (Ala>Ala)	2/9	Codon 147 GTT>ATT (Val>IIe)	1/12
	Deletion codon 304 – codon 337	1/9	Codon 225 GTT>GTG (Val>Val)	1/12
RA12	Codon 355 GCT>GCC (Ala>Ala)	1/12	Codon 213 CGA>CAA (Arg>Gln)	1/11
			Codon 213 CGA>CGG (Arg>Arg)	1/11
			Codon 269 AGC>AAC (Ser>Asn)	1/11
RA13	Codon 134 TTT>CTT (Phe>Leu)	1/12	None	0/11
	Codon 216 GTG>ATG (Val>Met)	1/12		
	Codon 249 AGG>GGG (Arg>Gly)	3/12		

Data presented as number of mutant subclones/total number of subclones analyzed. Nested PCR required for RA12 and RA13 FLS and RA10 – RA13 synovial tissues. ^a ^Recognized *p53 *polymorphism also detected in normal peripheral blood mononuclear cells (most probably germline).

**Table 3:** *p53 *mutation clusters identified in greater than one subclone isolated from rheumatoid arthritis (RA) fibroblast-like synoviocytes (FLS) or from synovial tissue

*p53 *mutation	Origin	Reference
Codon 138 GCC>GTC (Ala>Val)	Microdissected synovial tissue	[9]
Deletion codon 143 – codon 220	Microdissected synovial tissue	[9]
Codon 144 CAG>TAG (Gly>stop)	Microdissected synovial tissue	[9]
Codon 176 TGC>CGC (Cys>Arg)	Microdissected synovial tissue	[9]
Codon 213 CGA>TGA (Arg>stop)	Microdissected synovial tissue	[9]
Codon 245 GGC>GAC (Gly>Asp)	Cultured FLS	[23]
Codon 249 AGG>GGG (Arg>Gly)	Cultured FLS	Present study
Codon 300 CCC>TCC(Pro>Ser)	Microdissected synovial tissue	[9]
Codon 307 GCA>GCT (Ala>Ala)	Cultured FLS	Present study
Codon 321 AAA>GAA (Lys>Glu)	Cultured FLS	Present study
Codon 337 CGC>AGC (Arg>Ser)	Microdissected synovial tissue	[9]
